# Predicting Object Size from Hand Kinematics: A Temporal Perspective

**DOI:** 10.1371/journal.pone.0120432

**Published:** 2015-03-17

**Authors:** Caterina Ansuini, Andrea Cavallo, Atesh Koul, Marco Jacono, Yuan Yang, Cristina Becchio

**Affiliations:** 1 Department of Robotics, Brain and Cognitive Sciences, Fondazione Istituto Italiano di Tecnologia, Genova, Italy; 2 Centre for Cognitive Science, Department of Psychology, University of Turin, Torino, Italy; 3 Department of BioMechanical Engineering, Delft University of Technology, Delft, The Netherlands

## Abstract

Research on reach-to-grasp movements generally concentrates on kinematics values that are expression of maxima, in particular the maximum aperture of the hand and the peak of wrist velocity. These parameters provide a snapshot description of movement kinematics at a specific time point during reach, i.e., the maximum within a set of value, but do not allow to investigate how hand kinematics gradually conform to target properties. The present study was designed to extend the characterization of object size effects to the temporal domain. Thus, we computed the wrist velocity and the grip aperture throughout reach-to-grasp movements aimed at large versus small objects. To provide a deeper understanding of how joint movements varied over time, we also considered the time course of finger motion relative to hand motion. Results revealed that movement parameters evolved in parallel but at different rates in relation to object size. Furthermore, a classification analysis performed using a Support Vector Machine (SVM) approach showed that kinematic features taken as a group predicted the correct target size well before contact with the object. Interestingly, some kinematics features exhibited a higher ability to discriminate the target size than others did. These findings reinforce our knowledge about the relationship between kinematics and object properties and shed new light on the quantity and quality of information available in the kinematics of a reach-to-grasp movement over time. This might have important implications for our understanding of the action-perception coupling mechanism.

## Introduction

In everyday life, we reach for objects, grasp and manipulate them, and use them to act on other objects. All these actions are apparently very simple. Yet, this is not so [1]. Grasping requires coding of the object intrinsic properties (e.g., its size, texture, and weight) and extrinsic properties (e.g., orientation or spatial location), and the transformation of these properties into a pattern of distal movement (e.g., [2]). Traditionally, this pattern has been described by means of variables such as the maximum grip aperture (i.e., the largest distance reached between the thumb and the index finger during the approaching phase) and the peak velocity of the wrist (i.e., the highest velocity reached by the wrist during the approaching phase). For example, it is well known that for a given object shape and orientation, the maximum grip aperture is greater and occurs later when the to-be-grasped object is large compared to when it is small [3–5]. Furthermore, the peak velocity of the wrist occurs later during movement as the target size increases [6–7].

Kinematic landmarks like peaks (and their times of occurrence) provide an instantaneous snapshot of the hand kinematics at discrete points in time. Despite their usefulness in characterizing some aspects of the reaching to grasp behavior, however, they do not allow the shape of kinematic profiles that are functions of time to be compared. The motor configuration that is formed by the hand in contact with the object is the result of a motor sequence that begins well ahead of the grasping action itself. Participants do not mold their hand to the target object at the end of the reach. Rather, they *gradually* open their grip during the hand transport to achieve an aperture wider than the object to be grasped, and then, *gradually* close their grip to conform it to the size of the object [8].

The present study was designed to extend the characterization of the effects of object size to the temporal domain. To this aim, we computed the wrist velocity and the grip aperture throughout reach-to-grasp movements aimed at large versus small objects. To obtain a complete spatiotemporal description of grasping postural adjustments, we also considered how hand configuration varied over time. The majority of studies investigating the effect of object size have paid little attention to how the shape of the hand evolves gradually to conform to the contours of the object (for a critical review on this, please refer to [9]). In most cases, indeed, participants were instructed to use a precision grip with no regard given to the object’s size, and only the maximum grip aperture was measured. To provide a more complete and finer characterization of anticipative postural adjustments of the hand, we also considered the time course of finger motion relative to hand motion (‘local’ frame; see [10–11]).

In addition to classical statistical techniques, we used multivariate classification analysis to probe the discriminatory capacity of kinematic features over time. In particular, we used a support vector machine (SVM) algorithm to discriminate between reach-to-grasp movements towards large and small objects at different time intervals. The SVM algorithm works as a supervised learning model to find the optimal separating hyperplane between the data sets, i.e., the surface that best separates the data sets. The capacity of SVM to discriminate kinematics data in a binary task was reported previously (e.g., [12]). In the present case, the ability to classify the data sets correctly would indicate the presence of sufficient differences in the kinematics of grasping movements for targets of different sizes. This would lead to an above chance level (50%) prediction performance in the corresponding time interval.

## Methods

### Participants

Fifteen participants took part in the study. They had a mean age of 28 years (SD: 4.7; range: 23–35 years old; 8 females) and were all right handed, as measured by the Edinburgh Handedness Inventory [13], with normal or corrected-to-normal vision, and with no history of either psychiatric or neurological disorders. The experimental procedures were approved by local ethical committee (ASL 3 Genovese) and were carried out in accordance with the principles of the revised Helsinki Declaration (World Medical Association General Assembly, 2008). Written consent was provided by each participant.

### Apparatus and Procedures

Participants were seated on a height-adjustable chair with the elbow and wrist resting on a table, the forearm pronated, the right arm oriented in the parasagittal plane passing through the shoulder, and the right hand in a semi-pronated position, with the tips of the thumb and index finger on a tape-marked point. The angular position of the wrist was also controlled as to guarantee a repeatable start position across participants. The working space was set on the surface of a table (wide = 140 cm; deep = 70 cm) covered with a black cloth. Participants were asked to reach, grasp, lift, and move an object to an area (26 x 10 cm) located 50 cm to the left of the object’s initial position The target object could be either a grapefruit (referred to as ‘large object’) or a hazelnut (referred to as ‘small object’). The diameter of the large object was about 5 cm and its weight was equal to 354 g. The diameter of the small object was about 1.5 cm and its weight was equal to 2 g. Depending on condition, the large or the small object was placed on the table. The object, positioned at a distance of about 48 cm from the starting position, was aligned with the participant’s midline. The angle between the sagittal plane passing through the object and the hand start position was about 35°. Participants were asked to grasp the object at a natural speed using their right hand. The experimenter visually monitored the performance of each trial to ensure participants’ compliance to these requirements. Participants performed a total of 60 trials in 6 separate blocks of 10 trials (3 blocks of 10 trials for each of the two sizes). On average, the time between trials was 15 s. Blocks were presented in a pseudo-randomized order. The experimental session was preceded by a practice session to familiarize participants with the task (20 practice trials; 10 small object trials, 10 large object trials).

### Kinematics Recording

To track the kinematics of the hand, we used a near-infrared camera motion capture system (frame rate: 100 Hz; Vicon System). Eight cameras were placed in a semicircle at a distance of 1.5–2 m from the table on which the object was placed. Each participant was outfitted with lightweight retro-reflective hemispheric markers (4 mm in diameter). Markers were placed on the radial aspect of the wrist (rad), the metacarpal joint and the tip of the index finger (ind1 and ind3, respectively), the metacarpal joint of the little finger (lit1), the trapezium bone of the thumb (thu0) and the tip of the thumb (thu4). Additional markers (not used to compute the variables of interest) were placed on the metacarpal and proximal interphalangeal joints of the thumb, the proximal interphalangeal joint the index finger, and the proximal interphalangeal joint and the tip of the little finger. To set the tracking volume, the system was calibrated before data collection by first having the participants perform reaching and grasping movements as the experimenter adjusted the camera position, roll angle, zoom, focus, threshold, and brightness.

### Data Processing and Kinematics Variables

**Fig 1:**
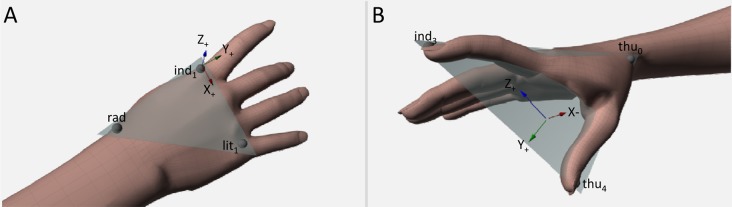
Lateral and frontal view of hand model. In Panel A, the local frame of reference (F_local_) determined by using the markers *rad* (placed on the radial aspect of the wrist), *ind1* (placed on the metacarpal joint of the index finger) and *lit1* (placed on the metacarpal joint of the little finger). *x-y* represents the metacarpal plane, *y-z* represents the sagittal plane. In Panel B, the finger plane defined as x, y and z components of the thumb – index plane defined as passing through the markers *thu0* (placed on the trapezium bone of the thumb), *ind3* (placed on the tip of the index finger), and *thu4* (placed on the tip of the thumb). Additional markers (not used to compute the variables of interest) were placed on the metacarpal and proximal interphalangeal joints of the thumb, the proximal interphalangeal joint the index finger, and the proximal interphalangeal joint and the tip of the little finger.

After data collection, each trial was individually inspected for correct marker identification and then run through a low-pass Butterworth filter with a 6 Hz cutoff. For data processing and analysis, a custom software (Matlab; MathWorks, Natick, MA) was used to compute the following variables:

- *Time of reach onset* defined as the first time point at which the wrist velocity crossed a 20 mm/s threshold and remained above it for longer than 100 ms;
- *Time of reach offset* defined as the time at which the wrist velocity dropped below a 20 mm/s threshold;
- *Movement Duration* defined as the time interval between reach onset and offset (ms);
- *Wrist Velocity* defined as the module of the velocity of the wrist marker (mm/sec);
- *Wrist Height* defined as the z-component of the wrist marker (mm);
- *Grip Aperture* defined as the distance between the marker placed on thumb tip and that placed on the tip of the index finger (mm) (see Fig. 1 – Panel B; *thu4* and *ind3*, respectively).

These variables were expressed with respect to the original frame of reference (i.e., the frame of reference of the motion capture system, termed as global frame of reference; F_global_). In addition, to provide a better characterization of the hand joint movements, a local frame of reference centered on the hand was established (i.e., F_local_; see [10–11] for a similar method). F_local_ had its origin in *ind1* marker (see Fig. 1 – Panel A). Vectors (*ind1* ˗ *lit1*) and (*ind1* ˗ *rad*) defined the metacarpal plane of the hand (refer to the colored triangle in Fig. 1 – Panel A). In this frame of reference, the x-axis had the direction of the vector (*ind1* ˗ *lit1*) and pointed ulnarly, the z-axis was normal to the metacarpal plane, pointing dorsally, while the y-axis was calculated as the cross-product of z- and x-axes, pointing distally (see Fig. 1 – Panel A).

Within F_local_ we computed the following variables:

g. *x-*, *y-*, *and z-thumb* defined as x-, y- and z-coordinates for the thumb with respect to *F*_*local*_ (mm);
h. *x-*, *y-*, *and z-index* defined as x-, y- and z-coordinates for the index finger with respect to *F*_*local*_ (mm);
i. *x-*, *y-*, *and z-finger plane* defined as x-, y- and z-components of the thumb-index plane, i.e., the three-dimensional components of the vector that is orthogonal to the plane. This plane is defined as passing through *thu0*, *ind3* and *thu4*, with components varying between +1 and ˗1 (please refer to Fig. 1 – Panel B). This variable provides information about the abduction/adduction movement of the thumb and index finger irrespective of the effects of wrist rotation and of finger flexion/extension.

For all trials, kinematics variables were expressed with respect to normalized (%) rather than absolute (ms) movement durations. This allows to compare kinematics variables of interest across experimental conditions at different epochs during reaching (from 10 to 100% of movement duration, at 10% intervals). This procedure was applied to allow comparison of hand postures across trials and participants.

### Design and Statistical Analyses

For the above kinematics variables, we analyzed the significance of differences between ‘object size’ (2 levels; small vs. large) and ‘time’ (10 levels; from 10 to 100% in 10 steps) using a within-subject multivariate analysis of variance (MANOVA) in order to protect the analyses from family-wise error inflation. The MANOVA was followed by separate ANOVAs on each of the dependent variables. Main effects were used to explore the means of interest (*post hoc t* test), and Bonferroni's corrections (α level < 0.05) were applied.

#### Classification analysis

To ascertain the predictive capability of kinematic features over time, we also employed a machine learning approach. Such an approach can evaluate the predictability of the data labels from the data features and can be extremely useful in the field of motor control, where it is necessary to comprehend the activities of a large number of variables (see [12, 14]). Specifically, here a support vector machine (SVM) algorithm was used to classify movements aimed at reaching large and small objects using kinematic parameters at different time points as features. An SVM, in essence, finds the solution to the following optimization problem:

$$\begin{array}{l} \;\;\underset{w,b,\xi }{\min}\;\frac{1}{2}w^{T}w+C\overset{l}{\underset{i=1}{\sum }}\xi_{i}\\ \text{subject to}\;\;y_{i}(w^{T}\phi \left(x_{i}\right)+b)\geq 1-\xi_{i}\\ \;\;\;\;\;\xi_{i}\geq 0,\;\forall i \end{array}$$

Where (x_i_,y_i_), *i = 1*,*2*,*3*.....*l*, is a training set of instance-label pairs (*x*_*i*_ ∈ *R* ^*n*^ and *y* ∈ {1: −1}^*l*^). Specifically, the algorithm tries to find an optimal separating hyperplane, defined by the equation *w* ^*T*^ *x* + *b* = 0 (where *w* is a normal vector and *b* is a scalar) that best separates the two classes. The parameters *w* and *b* are found by maximizing the distance (1/||*w*||) (equivalent to minimizing ||*w*||) between the plane and nearest data points of each class subjected to the constraint *y*_*i*_ (*w* ^*T*^ *x*_*i*_ + *b*) ≥1. Slack variables (*ξ*_*i*_) are introduced for handling and accounting for non-separable data. To penalize misclassification, a constant regularization parameter (C) is used.

Since it would be difficult to find the optimal hyperplane if the data are not linearly separable, a kernel function K (*x*_*i*_, *x*_*j*_) ≡ *ϕ*(*x*_*i*_)^*T*^ *ϕ*(*x*_*j*_) is often employed in SVM to project the data to a surrogate space. This permits the construction of a linear hyperplane between the two classes in feature space. Here, we use a radial basis function (*K*(*x*_*i*_, *x*_*j*_) = exp (−*γ||x*_*i*_−*x*_*j*_ *||* ^2^), where *γ* > 0) as the kernel for the SVM (where *γ* is the Gaussian kernel parameter). Using this kernel function, two parameters i.e., C and γ, need to be determined in the SVM [15]. We used a grid search to find optimized values of these two parameters.

All the trials from all the 15 participants were included in the classification analysis. Values deviating more than 2 SD from the mean for a particular participant for a specific object were treated as outliers (<6%) and replaced using Matlab File Exchange submission inpaint_nans (http://www.mathworks.com/matlabcentral/fileexchange/4551-inpaint-nans). This procedure interpolates and extrapolates based on sparse linear algebra and Partial Differential Equations (PDE) discretization. A default method was used to solve approximations to PDEs using least squares approach in case of interpolation, while for extrapolation a linear behavior was applied. Values deviating more than 2 SD from the mean at group level for a particular object (<5%) were replaced by a random value between the group mean and 1 SD. For the feature classification, open source SVM machine learning library (libsvm; http://www.csie.ntu.edu.tw/∼cjlin/libsvm/) was used.

Accuracy rates were computed from a ten-fold cross validation scheme in which values were averaged. A complete formulation of SVM can be found in a number of publications (e.g., [16–17]). In particular, a full description of the SVM algorithm in a binary task classification is provided by Tolambiya and colleagues [18].

Lastly, we used a criterion called F-score to evaluate the importance of kinematic features for object size prediction [19]. We calculated the discrimination ability of each kinematic feature and compared it to the overall discriminative power of all kinematic features. F-score is a simplified Fisher criterion, which is suitable to estimate the discriminative power of single features as well as of group of features (feature vectors). For single features, F-score was computed in the same way as the classic Fisher criterion [20]. For feature vectors, F-score was calculated as:

$$F=\frac{{\left\|{\vec{\mu }}_{+}-{\vec{\mu }}_{-}\right\|}_{2}^{2}}{tr\left(\Sigma_{+}\right)-tr(\Sigma_{-})}$$

where ${\vec{\mu }}_{+}$ and ${\vec{\mu }}_{-}$ are the means of feature vectors and Σ_+_ and Σ_−_ are the covariance matrices of feature vectors for class “*+”* and “-” respectively, *tr*() denotes the trace of a matrix and ||⋅||_2_ denotes the Euclidean norm. The numerator of the expression represents the variance between the classes, while the denominator denotes the variance within the classes. In this work, the feature vector is composed by all kinematic features for each class. To distinguish it from single feature F-scores, we called the overall F-score of all kinematic features F-group score.

## Results

The MANOVA revealed a significant main effect of both ‘object size’ [F_12,108_ = 126.375; *p* < .001] and ‘time’ [F_12,108_ = 33.488; *p* < .001]. Moreover, the MANOVA showed a ‘object size’ by ‘time’ interaction [F_12,108_ = 16.459; *p* < .001]. Similar effects were also reported when considering each dependent measure separately. In particular, repeated measures ANOVAs revealed a main effect of ‘object size’ for *Wrist Velocity* [F_1,14_ = 24.538; *p* < .001], *Grip Aperture* [F_1,14_ = 406.867; *p* < .001], *Wrist Height* [F_1,14_ = 345.491; *p* < .001], *x-index* [F_1,14_ = 136.174; *p* < .001], *y-index* [F_1,14_ = 231.573; *p* < .001], *z-index* [F_1,14_ = 183.383; *p* < .001], *x-thumb* [F_1,14_ = 90.569; *p* < .001], *y-thumb* [F_1,14_ = 130.878; *p* < .001], *z-thumb* [F_1,14_ = 22.363; *p* < .001], *x-finger plane* [F_1,14_ = 10.894; *p* < .01], *y-finger plane* [F_1,14_ = 9.775; *p* < .01] and *z-finger plane* [F_1,14_ = 67.300; *p* < .001]. In line with previous results (see [21] for a review), inspection of the main effect of ‘object size’ revealed that the *Grip Aperture* was larger (mean ± SE = 73.82 ± 2.20 mm vs. 42.17 ± 1.22 mm) and the *Wrist Velocity* was higher (555.74 ± 16.57 mm/s vs. 521.25 ± 13.28 mm/s) for the large compared to the small target object. In addition, the *Wrist Height* was higher (85.08 ± 3.53 mm vs. 68.76 ± 3.53 mm) for grasping movements aimed at the large rather than at the small object.

For the F_local,_ post hoc tests revealed that the index finger pointed more ulnarly (*x-index* equal to −5.99 ± 2.32 vs. 3.87 ± 2.17), was less distal (*y-index* equal to 54.76 ± 2.09 vs. 69.32 ± 1.92), and extended more in the palmar direction (*z-index* equal to −73.32 ± 2.28 vs. −51.48 ± 3.21) when the to-be-grasped object was small rather than large. The thumb pointed more radially (*x-thumb* equal to −15.17 ± 1.60 vs. −2.194 ± 1.78), was more distal (*y-thumb* equal to 6.21 ± 1.94 vs. 14.85 ± 1.71), and extended less in the palmar direction (*z-thumb* equal to −82.40 ± 1.44 vs. −79.22 ± 1.50) when the object was large than when it was small. Finally, inspection of the *finger plane* components (*x-*, *y-*, and *z-finger plane*) indicated that the *x-finger plane* passing through the thumb and the index was more normal (−.963 ± 007 vs. −.948 ± .006), the *y-finger plane* and the *z*-*finger plane* were smaller (.073 ± 0.37 vs. .153 ± .038 and .051 ± .021 vs. .190 ± .022) for the small object in comparison to the large object.

**Fig 2:**
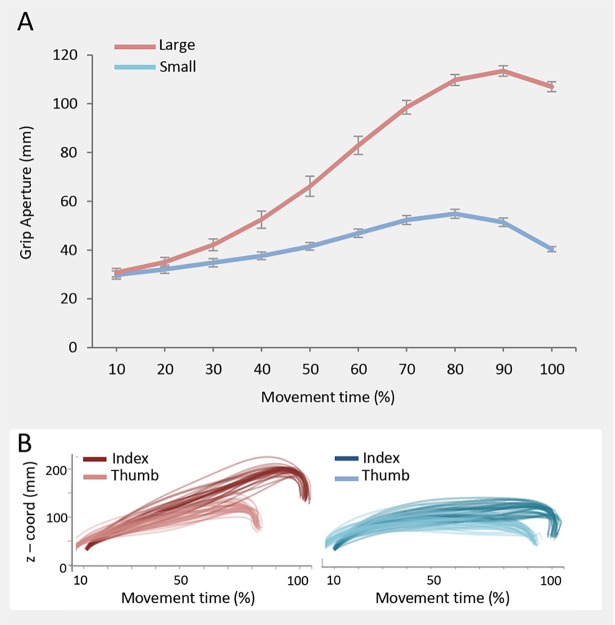
Grip aperture over time for both small and large object. In Panel A, grip aperture (mm) over time for both the small and the large object. Error bars represent standard errors. In Panel B, example from a representative participant of the index – thumb distance over time for both large (red) and small (light blue) object. Each line represents one trial.

**Fig 3:**
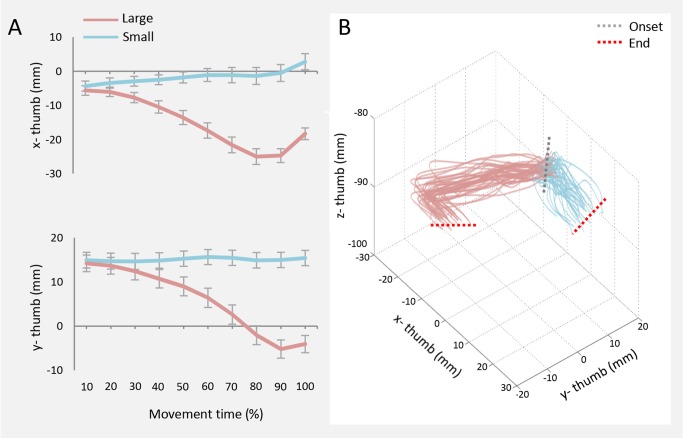
X, y, and z-thumb over time for both small and large object. In Panel A, *x-* and *y-thumb* coordinate over time for both small and large object. Error bars represent standard errors. In Panel B, example from a representative participant of a tridimensional representation of the *x-*, *y-* and *z-thumb* coordinate for both large (red) and small (light blue) object. Each line represents one trial.

**Fig 4:**
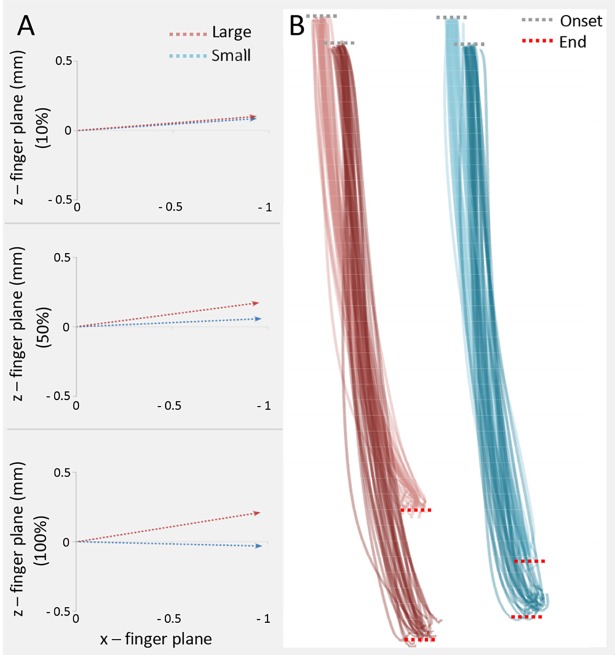
X- and z- components of finger plane over time for both small and large object. In Panel A, *x* and *z* components of finger plane at 10%, 50% and 100% of the movement time for both small and large object. In Panel B, example from a representative participant of the thumb and index finger tips position during the action unfolding.

Remarkably, the repeated measure ANOVAs also yielded a main effect of ‘time’ (F_9,126_ ranging from 4.983 to 246.750; *p*_s_< .001) as well as a significant ‘object size’ by ‘time’ interaction (F_9,126_ ranging from 10.698 to 257.009; *p*_s_< .001) for all the dependent measures. Post hoc comparisons using Bonferroni’s correction revealed that from 10% up to 40% of the reach-to-grasp movement, the *Wrist Velocity* was higher for small object compared to large object movements (*p*_s_ ranging from .015 to .037). In contrast, from 60% to 100% of the movement time, the *Wrist Velocity* was higher for large than for small object movements (*p*_*s*_< .01). No statistical differences were found at 50% of the action (*p* = .951) for this variable. With respect to the *Grip Aperture*, post hoc inspection of the interaction revealed greater modulation for the large than for the small object over time. No other sources were found indicating that, since the very beginning of the movement, the *Grip Aperture* was greater for large than for small objects (see Fig. 2). Furthermore, the *Wrist Height* was greater for large in comparison to small object movements from 40% up to the end of the reach-to-grasp movement (*p*_s_ ranging from .000 to .022), while no differences were evident from 10% to 30% of the movement. For the index finger, a significant difference between the two objects was found for the *x-index* throughout the entire duration of the movement (*p*_*s*_ ranging from .000 to .028), and from 20% to 100% for both the *y*- (*p*_s_ ranging from .000 to .012) and *z*-*index* (*p*_s_ ranging from .000 to .030). For the thumb, both *x*-*thumb* and *y*-*thumb* significantly differed between objects for the whole movement duration (please refer to Fig. 3). Similarly, the *z*-*thumb* was sensitive to the size of the target, although the difference between small and large was evident only from 50% up to the end of the movement. A profile inspection of the *x*-, *y*-, and *z*-*thumb* revealed that, when the to-be-grasped object was small, this digit exhibited a reduced modulation in comparison to when the object was large (Fig. 3). This was evident for the entire duration of the reaching movement, suggesting that the stability of the thumb relative to the object was greater for the small target compared to the large target [22]. Finally, for the *finger plane*, post hoc contrasts revealed that the *x-finger plane* was significantly more normal with respect to F_local_ for small than for large object movements from 60% to 90% of the action (*p*_*s*_ ranging from .000 to .024) (Fig. 4), while the *y*- and the *z*-*finger plane* were different from 60% to 100% (*p*_*s*_ ranging from .001 to .011) and from 30% to 100% of the movement (*p*_*s*_ ranging from .000 to .007), respectively.

### Classification analysis

**Fig 5:**
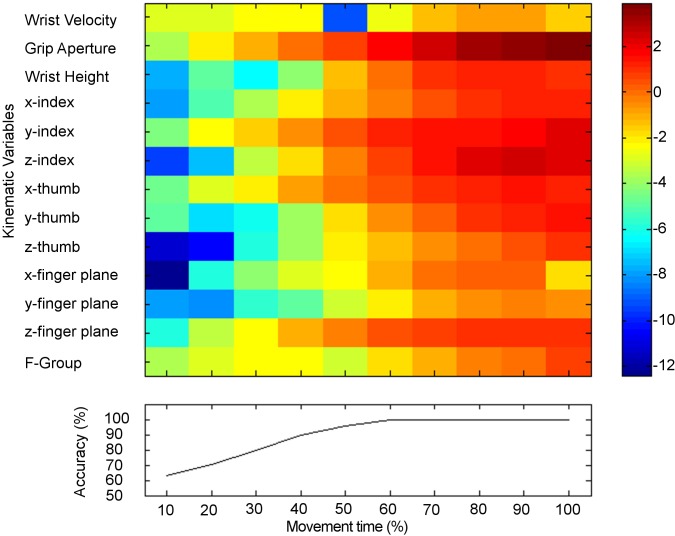
Predictability values matrix. Predictability and discriminability of kinematics variables over time. Graphical representation of F-scores of kinematics variables (heat map) and accuracies (line graph) from classification analysis. Please note that F-group value is a combined F-score of all the variables at a given time interval.

As displayed in Fig. 5, early on in the movement, the ability to predict the size of the target to be grasped was above chance level (63.14% at 10% of movement, 70.49% at 20%). Accuracy rates increased as time progressed (95.81% at 50% of movement), achieving ∼100% of accuracy at 60% of the movement (accuracy rates ranging from 99.47% at 60% to 99.97% at 100% of movement duration). Similarly, we found a gradual increase of F-scores across time intervals (Fig. 5). In particular, F-scores increased gradually for *Grip Aperture*, *y-index*, *x-thumb*, and *z-finger plane* from 20% of movement duration to the end of the movement. For *Wrist Height*, *x-index*, *z-index*, *y-thumb*, a consistent increase in F-scores was observed from 50% to 100% of the movement duration. Only for *Wrist Velocity*, F-score decreased at 50% of the movement duration (please refer to Fig. 5).

The F-group score increased monotonically as time progressed. Of interest, an inspection of this value also revealed an unequal contribution from the underlying features, suggesting that some kinematics features underwent more change during movements towards targets of different sizes. In this regard, it is worth noticing that, for instance, F-scores for *Grip Aperture* were higher than F-group scores throughout the entire movement duration (see Fig. 5).

## Discussion

The ability to generate a successful grasping movement is dependent on extracting intrinsic properties from the intended target object. For example, to successfully reach, grasp, and lift an object, in addition to the processing of the object location, it is paramount to encode the object size and shape, as well as other intrinsic properties such as fragility, texture, and weight. It is already well known that all these properties influence grasping kinematics [9]. To date, however, only a few studies have characterized the effect of object properties on the grasping behavior over time.

Evidence that specification of some movement parameters evolves in a time-dependent and continuous fashion during the reaching was first provided by Santello and Soechting [23]. They asked participants to reach and grasp differently shaped objects. Critically, the information transmitted by hand posture about object shape was still incomplete at the time of maximum grip aperture (at about 50% of movement duration), suggesting the hand was only gradually molded to conform to the shape of the object to be grasped [23]. Because other parameters of the motion, such as movement direction, are fully specified early on in a movement (e.g., [24]), these results were interpreted to suggest that the specification of diverse aspects of a movement evolves with different time courses. This conclusion was further supported by subsequent studies in which gradual preshaping of the hand to the object contours was investigated as function of task [25–26] and sensory feedback [27–28], in both healthy and neurologically impaired individuals [29–32].

The present results add to this body of research showing that the specification of kinematic variables evolved in parallel but at different rates in relation to object size. While some variables such as grip aperture and wrist velocity were specified early on in the movement, already discriminating between the sizes of the target at 10% of movement duration, others, such as wrist height, showed little modulation until 60% of movement duration. Interestingly, differences in the pattern of temporal modulation were also observed at the level of finger joint posture. In particular, both index finger and thumb exhibited a kinematic attunement to the size of the target from the beginning of the movement (between 10% and 20% of the movement duration) up to the moment of hand-object contact. For the finger plane, a similar modulation was observed later, between 30% and 60% of the movement time, suggesting that this parameter evolved at a slower rate.

### Implications for action observation

Taken together, these findings suggest that while some parameters are already specified at the time of movement initiation, others evolve gradually over the whole movement duration. This timing information may be useful not only for understanding the mechanisms subtending execution, but also for better comprehending action observation. Observing someone else perform an action recruits a set of sensorimotor brain regions that are active during movement execution (mirror response; [33]). The precise timing of this internal simulation and its relation to the timing of the external action, however, are poorly understood [34].

To date, the majority of the studies investigating the mirror response to observed actions have focused on specific kinematics landmarks such as the maximum grip aperture and the peak velocity of the wrist to characterize observed movements (e.g., [35–36]). These parameters provide a snapshot description of the observed movement at a single time point during reach, i.e., the maximum within a set of value. To ascertain the temporal course of the mirror response, however, we need to know how hand kinematics evolves throughout movement duration: i) ‘when’ diverse aspects of the movement are specified, e.g., when kinematics discriminate correctly the properties of the target; ii) and the time courses at which diverse kinematics parameters evolve.

Extant research suggests that modulation of corticospinal excitability is not an immediate mirror response to the visual stimulus (e.g., the appearance of the maximal finger aperture), but rather reflects a prediction of the movement to occur [37–40]. Knowing how movement kinematics is modulated during reaching may prove critical to determine the time at which we would expect to see a specific modulation to the observed action, as well as to understand better the temporal matching between the observed and the executed action.

Importantly, this approach may also help to identify the contribution of specific discriminative features over time. As different kinematics features contribute differently to the specification of the upcoming state, it is plausible that, when asked to predict how an action would unfold, observers rely on a subset of correlated variable to predict how the action will unfold. We are tempted to speculate further that this may lead observers to adopt a “dynamic” strategy: when the level of predictability of different features varies over movement duration (as in the present case), observers may exploit different features at different time points, taking into account those features that at each time interval best predict the action outcome.
